# Intimate partner violence against women in eastern Uganda: implications for HIV prevention

**DOI:** 10.1186/1471-2458-6-284

**Published:** 2006-11-20

**Authors:** Charles AS Karamagi, James K Tumwine, Thorkild Tylleskar, Kristian Heggenhougen

**Affiliations:** 1 Department of Paediatrics and Child Health, Makerere University, P.O.Box 7072, Kampala, Uganda; 2 Clinical Epidemiology Unit, Makerere University, P.O.Box 7072, Kampala, Uganda; 3 Centre for International Health, University of Bergen, Armauer Hansen Bldg, N-5021 Bergen, Norway; 4 Department of International Health, Boston University School of Public Health, 715 Albany Street, T4W, Boston, MA 02118, USA

## Abstract

**Background:**

We were interested in finding out if the very low antenatal VCT acceptance rate reported in Mbale Hospital was linked to intimate partner violence against women. We therefore set out to i) determine the prevalence of intimate partner violence, ii) identify risk factors for intimate partner violence and iii) look for association between intimate partner violence and HIV prevention particularly in the context of the prevention of mother-to-child transmission of HIV programme (PMTCT).

**Methods:**

The study consisted of a household survey of rural and urban women with infants in Mbale district, complemented with focus group discussions with women and men. Women were interviewed on socio-demographic characteristics of the woman and her husband, antenatal and postnatal experience related to the youngest child, antenatal HIV testing, perceptions regarding the marital relationship, and intimate partner violence. We obtained ethical approval from Makerere University and informed consent from all participants in the study.

**Results:**

During November and December 2003, we interviewed 457 women in Mbale District. A further 96 women and men participated in the focus group discussions. The prevalence of lifetime intimate partner violence was 54% and physical violence in the past year was 14%. Higher education of women (OR 0.3, 95% CI 0.1–0.7) and marriage satisfaction (OR 0.3, 95% CI 0.1–0.7) were associated with lower risk of intimate partner violence, while rural residence (OR 4.4, 95% CI 1.2–16.2) and the husband having another partner (OR 2.4, 95% CI 1.02–5.7) were associated with higher risk of intimate partner violence. There was a strong association between sexual coercion and lifetime physical violence (OR 3.8, 95% CI 2.5–5.7). Multiple partners and consumption of alcohol were major reasons for intimate partner violence. According to the focus group discussions, women fear to test for HIV, disclose HIV results, and request to use condoms because of fear of intimate partner violence.

**Conclusion:**

Intimate partner violence is common in eastern Uganda and is related to gender inequality, multiple partners, alcohol, and poverty. Accordingly, programmes for the prevention of intimate partner violence need to target these underlying factors. The suggested link between intimate partner violence and HIV risky behaviours or prevention strategies calls for further studies to clearly establish this relationship.

## Background

The World Health Organization defines intimate partner violence against women as "the range of sexually, psychologically and physically coercive acts used against adult and adolescent women by current or former male intimate partners"[1]. Intimate partner violence is an important problem because it is global, violates fundamental human rights of women, and is a major public health problem [2-9]. Intimate partner violence is the most common form of violence against women[1]. Worldwide, the prevalence of lifetime intimate partner violence has been reported to be 10% to 71% of women in marriage or current partnerships [10-18]. In sub-Saharan Africa, the reported prevalence of intimate partner violence ranges from 20% to 71%[14,18-21]. However, the prevalence of intimate partner violence is believed to be under-estimated because of under-reporting and lack of standardization of methods[1].

Intimate partner violence is associated with demographic, socio-economic, socio-cultural, and lifestyle factors. Higher age and numbers of children of women seem to be associated with a reduced risk of violence[17,22], while poverty and low education of male partners seem to be associated with increased risk of violence[10,13,16]. The relationship between the status of women (education, autonomy, control of resources) and intimate partner violence is less clear. Some studies report increased risk of violence [23] while others report decreased risk of violence with higher status of women[13,17,22].

Gender inequality, infidelity, and polygamy have been associated with increased risk of violence in South Africa[19]. In some societies, intimate partner violence may be perceived as a sign of love[19]. Marital conflict seems to be consistently associated with intimate partner violence [19], and several studies have demonstrated an association between use of alcohol or drugs and intimate partner violence [19-22]. Findings that children who witness family violence are more likely to be perpetrators or victims of violence in adulthood suggest that intimate partner violence may be intergenerational[10,19,24].

Intimate partner violence has also been linked to HIV infection. Some studies suggest that intimate partner violence increases the risk of HIV infection because of sexual violence[18,20,25-28]. The fear of intimate partner violence may also decrease HIV prevention behaviour and thus increase the risk for HIV infection[20,21,29]. Other studies suggest that HIV infection, whether in the woman or her partner, increases the risk of intimate partner violence because of refusal of sex or disclosure of HIV results[14,15,30]. Similarly, women's perceptions of their male partners' risk of HIV are also associated with intimate partner violence[14]. Finally, intimate partner violence and HIV infection may be associated because women with or at risk of HIV infection come from populations that are also at risk for intimate partner violence[20,31,32].

We were particularly interested in finding out if the very low antenatal VCT acceptance rate reported in Mbale Hospital was linked to intimate partner violence. We therefore conducted a community-based study in Mbale District, Uganda so as to i) determine the prevalence of intimate partner violence, ii) identify risk factors for intimate partner violence and iii) look for association between intimate partner violence and HIV prevention particularly in the context of the prevention of mother-to-child transmission of HIV programme (PMTCT).

## Methods

Mbale District is situated in eastern Uganda and borders the Republic of Kenya and Mt Elgon to the east. It has a population of over 720 000 of which 90% is rural and predominantly *Bagisu *who speak *Lumasaba*[33]. The literacy rate is 64 and 49 percent for males and females, respectively[34]. The prevalence of HIV was 5.6 percent in 2003[35]. The main economic activity is subsistence farming. Mbale District is divided into 4 counties namely Bubulo, Bunghoko, Manjiya, and Mbale town[33].

The study was conducted as part of the "Essential Child Health and Nutrition Project in Uganda", a collaboration between the Department of Paediatrics and Child Health, Makerere University and the Centre for International Health, Bergen University. The field site for the Project is Mbale District. We selected Mbale town and Bungokho county because they were in the field site of the project. The study consisted of a cross sectional household survey of women with infants, and focus group discussions with women and men. Sampling for the household survey was based on the WHO/EPI cluster survey method. We randomly selected 68 villages or wards (urban administrative units) each comprising of about 300 households within the county or town. Mbale town was intentionally over-sampled because of its denser population. Each village or ward constituted a cluster. With the help of local officials, we identified the centre of each cluster. At the centre of the cluster, a bottle was spun on the ground and the direction in which the top of the bottle pointed was taken to be the direction of the survey. The households between the centre and boundary of the cluster were identified and the first household was randomly selected. The second household was defined as the one nearest the first household moving in the chosen direction. Subsequent households were selected in a similar manner and at the boundary of the cluster, the interviewers turned clockwise and continued to select households until a total of seven households were identified. Only households that fulfilled the selection criteria were selected.

Participants were women aged 18 years and above and who had children aged one year or less. The women resided in the selected households in Mbale town or Bungokho county and consented to participate in the study. After consenting, the women were interviewed in their homes.

The instrument used to collect data was an interviewer-administered questionnaire with open ended and closed ended questions. It included 66 items on socio-demographic characteristics of the woman and her husband, antenatal and postnatal experience related to the youngest child, antenatal HIV testing, perceptions regarding the marital relationship, and intimate partner violence. Women were asked about their experience of male against female intimate partner violence involving their husbands over the past 12 months and ever. Women were asked whether their husbands had ever threatened them with a weapon (and the type of weapon used), kicked, bitten or hit them, or sexually coerced them (Table 1). Lifetime intimate partner violence was defined as lifetime occurrence of any form of intimate partner violence. A variable of household socio-economic status was developed by use of principal components analysis with variables on asset ownership (bicycle, radio, television, motorcycle, car/truck, land), materials of the dwelling structure (floor, wall, roof) and ownership of poultry and animals. We used principal components analysis to divide the households into quintiles of socio-economic status, with (1) poorest and (5) least poor. We categorised quintiles 1–3 as "poorest" and 4–5 as "least poor" which suggests that the population was generally poor.

We followed guidelines established by the World Health Organization for the collection of sensitive information on intimate partner violence[36]. We recruited 12 research assistants who were fluent in English and the local language *Lumasaba *and had experience in data collection. We trained them in sampling, interview technique, and ethical issues, emphasising the importance of safety of the participants and interviewers, minimization of under-reporting of sensitive information particularly on intimate partner violence, and confidentiality. We conducted a pilot survey and used the lessons to revise the procedures and instruments of the survey. The survey was conducted with the support of the local officials who assisted in the sampling procedures and introduction of the survey team to household members. The research assistants worked in pairs of a female and a male, and a pair interviewed seven respondents in one cluster in one day. The lead interviewer was a female and the role of the male was mainly to ensure the privacy of the interviews and also the safety of the participants and interviewers. The interviews were conducted in the privacy of the women's homes but away from husbands, relatives, friends or local officials. The houses were generally close together and interviews were conducted daily between 8 am and 6 pm. The research assistants were in direct communication by mobile telephone with a supervisor who could physically reach them in less than an hour.

We based our sample size calculation on estimation of the prevalence of intimate partner violence in rural and urban women in Mbale. We used an expected proportion of intimate partner violence of 0.15 and a total width of 0.10. We set the confidence level at 95% and incorporated a design effect of 2.0 because of the cluster design. We increased the sample size by 20% to cater for problems that might occur in recruitment. The estimated sample size was 476. Since 19 women were not available for interview, and we failed to get replacements for them, the final sample size was 457 women. All the women that were sampled agreed to participate in the study and thus there were no refusals. Quantitative data was entered into EPIDATA and then exported to Stata version 8.0 for analysis that adjusted for the design effect. We used the following dependent variables in our analysis; lifetime intimate partner violence, antenatal attendance, HIV test during last pregnancy, delivery in hospital, HIV talk with husband, and use of condom with husband. We performed bivariate analysis between each dependent variable and all the independent variables including the socio-demographic variables of the women and men. We then performed multiple logistic regressions for each dependent variable controlling for all the independent variables.

There were eight focus group discussions, four for women and four for men. Two focus group discussions, one for women and one for men, were held in each of the three sub-counties of Bungokho county (Namanyonyi, Bufumbo and Nakaloke), and in Namatala zone of Mbale town. The participants were married women with at least one previous delivery or married men. With the help of local officials, we recruited the participants purposively from each sub-county. We selected participants who had not been involved in the survey, of different backgrounds, not related, and not relatives of the local officials. The mobilization was very good and more people than were required turned up. We purposively selected 12 participants for each FGD, for a total of 96 participants. The participants were aged 18 to 60 years and were mainly *Bagisu*. Most of the participants had less than eight years of education and were subsistence farmers.

The discussions were held in the community and in privacy. Two female research assistants conducted the focus group discussions for women while two male research assistants conducted the focus group discussions for men. In each focus group discussion, one research assistant was the moderator while the other was a recorder to take notes and operate the tape-recorder. The moderator guided the discussion using an interview guide that covered the occurrence of intimate partner violence, its causes and relation to alcohol, polygamy, drugs, and HIV. The interview guide was flexible to allow related but unforeseen issues to be discussed as well. The discussions were conducted mainly in *Lumasaba*. Most of the discussions were very lively and participants spoke openly. The focus group discussions lasted between two to three hours. The data was tape-recorded, transcribed and then coded using the qualitative programme OpenCode. Themes were identified and used to complement the findings from the survey.

Institutional permission to conduct the study was obtained from Makerere University Faculty of Medicine Research and Ethics Committee and informed consent was obtained from all participants in the study.

## Results

### Socio-demographics, health utilization and psychosocial factors

Our sample consisted of 457 women (Table 2). Over half the women were rural (73%), aged less than 25 years of age (52%), Muslim (61%), and married (91%). The majority of the women were multiparous (78%), had less than 8 years of education (71%), and worked in agriculture (88%). Most women had attended antenatal clinic during the most recent pregnancy (97%) but only 47% delivered in a health unit. Most women had ever discussed HIV with their husbands (55%) but few had ever used condoms with their husbands (22%) or tested for HIV (10%) during the most recent pregnancy. Most women were satisfied with their marriages (76%); 35% reported that the husband had other sexual partners while 5% had ever had another sexual partner during their current relationship.

### Prevalence of intimate partner violence against women

The prevalence of lifetime intimate partner violence was 54% (Table 3). Lifetime physical violence and sexual coercion contributed most to intimate partner violence. The prevalence of physical violence in the past year was 14%. Women and men in focus groups confirmed that intimate partner violence was very common. Whereas women reported that quarrelling was most frequent, and that physical and sexual violence often occurred together, the men emphasised only the physical violence. The most frequently used weapons were *pangas *(large bush knife) (36%) and sticks (27%) (Table 4).

### Factors associated with intimate partner violence against women

On logistic regression model using intimate partner violence as the dependent variable, higher education of women (OR 0.3, 95% CI 0.1–0.7) and marriage satisfaction (OR 0.3, 95% CI 0.1–0.7) were associated with lower risk of intimate partner violence, while rural residence (OR 4.4, 95% CI 1.2–16.2) and the husband having another partner (OR 2.4, 95% CI 1.02–5.7) were associated with higher risk of intimate partner violence (Table 2). There was no association between intimate partner violence and antenatal attendance (OR 0.7, 95% CI 0.2–2.8), HIV test during last pregnancy (OR 1.8, 95% CI 0.6–5.3), delivery in a health facility (OR 0.7, 95% CI 0.4–1.2), HIV talk with husband (OR 1.6, 95% CI 1.0–2.6) or use of condom with husband (OR 1.2, 95% CI 0.7–2.3) when these variables were treated as dependent variables in logistic regression models.

### Association between sexual coercion and physical violence

There was a strong association between sexual coercion and lifetime physical violence (OR 3.8, 95% CI 2.5–5.7); physical violence in the past year (OR 3.7, 95% CI 2.1–6.6); threat with weapon (OR 3.0, 95% CI 1.6–5.7); and kicked, bitten or hit (OR 3.3, 95% CI 1.9–5.5) (Table 5). Women in the focus groups confirmed that physical violence was in most cases accompanied by sexual coercion. However, the overwhelming view among men was that "*my own wife cannot refuse me sex*" (FGD, Male, Namanyonyi) implying that sex within marriage cannot be coerced sex and therefore there is no marital rape.

### Reasons for intimate partner violence against women

According to the survey, the most cited reasons for intimate partner violence were the husband had another partner (25%), the woman neglected housework (14%), or she went out without permission or returned home late (14%)(Table 6). The focus groups reported that intimate partner violence was common among the youth and in polygamous marriages because of unequal love, neglect and jealousy.

" *You go for kadodi (circumcision dance) and when you return he beats you*" (FGD, Female, Namanyonyi)

"*You cannot stop them loving other women but we fight them (other women) and even bite their ears off and burn them with acid*" (FGD, Female, Namatala)

According to the focus groups, alcohol was considered to be a major cause of intimate partner violence. Other reasons cited included poverty, arguments over money, poor communication, disobedience, disrespect or infidelity by the wife, and the husband's harshness, late-coming, chewing of *khat *(*Catha edulis *is a widely used plant with psycho-stimulatory effects) or wanting to separate.

"*You ask him to buy something for home and he instead abuses you and beats you*" (FGD, Female, Bufumbo)

"*You grow your food and sell it. That very day he collects the money saying it is his land*" (FGD, Female, Bufumbo)

"*I come home and she has not cooked for my mother? As a man I must beat her*" (FGD, Male, Nakaloke)

Sexual problems were also causes of intimate partner violence. While the women mentioned lack of romance, passive sex and sexual coercion, men mentioned refusal of sex by women and sexual weakness of men as causes of intimate partner violence. On the question of whether intimate partner violence was a sign of love, most women disagreed.

"*A man who beats me does not love me*" (FGD, Female, Namanyonyi)

However, some women and men were of the view that intimate partner violence was sometimes beneficial because:

"*if the man loves you he beats you to shape your bad behaviour *" (FGD, Female, Nakaloke)

"*he loves you very much, he even brings meat the day after beating you*" (FGD, Female, Namatala)

### Intimate partner violence against women and HIV infection

According to the focus groups, men usually react violently when women go for HIV testing; disclose HIV test results or request to use condoms. Men perceive these situations as evidence of "*prostitution*" and therefore "*AIDS*" in the women (FGD, Male, Namatala). Furthermore, men abhor using condoms and prefer "*flesh*" (unprotected sexual intercourse)(FGD, Male, Bufumbo). It was also reported that men secretly puncture the condoms so that they can impregnate the women.

"*he can ask me why I went for a (HIV) test and call me a prostitute and beat me*" (FGD, Female, Nakaloke)

"*men never allow us to use condoms, if we suggest they beat us*" (FGD, Female, Nakaloke)

## Discussion and conclusion

Our survey included only women who had children aged one year or less and may not have been representative of all married women in the study area. However, selection bias may have been minimal considering that the survey findings were supported by the focus groups that involved a wider selection of married women. Muslims dominated our sample with a proportion of 62% compared to 12% in the general population of Uganda. However, religion was not associated with intimate partner violence.

Intimate partner violence is a sensitive subject that is often hidden from society because of shame [1,37]. In addition, the definition of intimate partner violence and in particular emotional and sexual violence is very challenging [1,37,38]. When does sexual intercourse between husband and wife become sexual coercion? The disparity between the views of the women and men in our study on sexual coercion clearly highlights the difficulty of distinguishing marital sexual intercourse from sexual coercion [37]. Furthermore, our study focussed on intimate partner violence perpetrated by men against women and therefore may have missed violence from other origins for example the women. It is thus possible that problems related to the measurement of intimate partner violence may have introduced bias in our study although we took precautions to minimize it. A major limitation of the study was the sample size. Due to the very low prevalence of HIV testing among women, our sample was too small to detect significant association between HIV testing and intimate partner violence especially after adjusting for the cluster effect. Finally, our study was cross sectional and it is impossible to infer a causal relationship between the variables. Notwithstanding these limitations, our population-based study has important findings on intimate partner violence and its implications for the prevention of HIV.

The prevalence of intimate partner violence against women in our study was high. However, the true prevalence of intimate partner violence is likely to be even higher since we did not measure emotional violence, and because of the lower representation of rural women in the study compared to the population of Mbale district. Our prevalence of 37% for physical violence is within the reported global range of 10% to 71% while the prevalence of sexual violence of 37% is also within the reported range of 5.9% to 59% [1,18]. The prevalence of physical violence in our study lies between 30% and 40% reported in two population-based studies from Uganda [14,39]. It is however lower than was reported in Zimbabwe (40%) [20] but higher than was reported in Rwanda (20%) [21] and South Africa (25%)[19]. Our view is that the observed differences in prevalence between the studies could partly be explained by differences in the definition of intimate partner violence by the investigators as well as the respondents.

Rural women had a four-fold increase in the frequency of intimate partner violence compared to urban women. Our findings are similar to reports from many countries[18] but contrast with results from the Philippines that found a lower frequency of intimate partner violence among rural women[40]. However, the same study reported that the more domains of decision-making men dominate, the greater the physical violence against women. Rural communities are usually more conservative and the bedrock of the socio-cultural values of traditional societies. These socio-cultural values define the gender norms of women and men including power, roles, responsibilities and obligations. The traditional social-cultural values typically promote an imbalance in power between women and men, with women being in a subordinate position[20]. This imbalance in power contributes to greater intimate partner violence among rural women[1,18]. Intimate partner violence is a result of the low status of the women and because violence within marriage is widely tolerated[18,20]. Furthermore, in rural communities with rigid cultural norms, greater autonomy of women may destabilize marital relations and thus precipitate more violence[18,41].

Less educated women were twice as likely to experience intimate partner violence compared to the more educated women, findings that are consistent with the literature[13,14,19,28]. However, our findings appear to contradict other reports that more educated women are at increased risk of physical and sexual violence[17,22]. This apparent conflict may be explained by the suggestion that the relationship between empowerment (including education) and intimate partner violence is curvilinear. Greater empowerment reduces the risk of violence up to a point, beyond which the risk increases before levelling off as empowerment becomes protective[19,40]. It has also been suggested that the relationship between education and intimate partner violence may be context specific[41]. Educated women in more conservative settings experience greater intimate partner violence while educated women in less conservative settings experience less intimate partner violence. Overall, women's education was protective against intimate partner violence since most of the women were at the lower end of the education spectrum. The significance of our findings is that strategies aimed at promoting education of the girl-child and literacy programmes for women are likely to be beneficial in the prevention of intimate partner violence.

Women who reported dissatisfaction with their marriages were more than twice as likely to experience intimate partner violence as women who expressed satisfaction. Our findings are consistent with previous reports that marital discord is the most consistent marker of intimate partner violence[19]. Level of satisfaction and intimate partner violence may be causally related or they may both be effects of common underlying factors such as the intensity and quality of communication between husband and wife, polygamy, adultery, neglect and others [42,43]. Inquiry about marriage satisfaction could possibly be used as an entry point to discuss the more sensitive issue of intimate partner violence.

Women who experienced physical violence were three times more likely to be sexually coerced than those who did not experience violence, findings that were confirmed by the women in the focus groups. Ellsberg et al reported that 33% of beatings in Nicaragua were commonly accompanied by forced sex[44]. According to the women in the focus groups, sexual coercion is usually part of the punishment meted out by the husband on the wife. Some women also said that when a woman refuses sex, the husband first beats her up before sexually coercing her[26]. However, some women suggested that forced sexual intercourse following physical violence is culturally prescribed or an attempt by the husband at reconciliation. In general, men in the focus groups downplayed the magnitude of sexual violence and even suggested that women enjoy forced sexual intercourse, a view that was supported even by some women. The importance of these findings is that strategies aimed at preventing intimate partner violence must target the negative attitudes regarding marital sex and intimate partner violence.

Thirty five percent of women reported that the husband had another sexual partner, a figure that also included polygamous marriage. Polygamy is widespread in Ugandan societies with reports of 1 in 3 women married in a polygamous union[34]. Women in the focus groups reported that the husband having another sexual partner was the most common cause of intimate partner violence because of unequal love, jealousy, and neglect. On the other hand, a woman who suspects that the husband has another sexual partner may become abusive and disrespectful because of jealousy. In this setting, intimate partner violence is likely to occur. Although the men downplayed the role of multiple female partners in intimate partner violence, clearly this is one of the problems fuelling it. The problem of multiple partners is also important because it is a barrier to safe sexual behaviour since women are not in a position to negotiate for safe sex[45].

The reasons for intimate partner violence are strikingly similar to reasons reported in the literature and differences exist mainly in the relative magnitude of the reasons[14,20,26,39,42]. Although only 5% of the women in the survey attributed intimate partner violence to alcohol, participants in the focus groups reported that consumption of alcohol, mainly by men but also by women, was an important reason for intimate partner violence[19,26,37,40,42]. Koenig et al reported that women whose partners drink before sex experienced risks of violence almost five times higher than women with non-drinking partners[14]. Consumption of alcohol is widespread in Uganda and is particularly important not only because it contributes to intimate partner violence but it is also a major barrier to sexual behaviour change[45]. The chewing of *khat *was also said to be a cause of men's "*madness*" leading to intimate partner violence. In recent years, Uganda has experienced an upsurge in the cultivation and trafficking of both *khat *and marijuana and this may increase the level of intimate partner violence especially in urban areas.

Poverty was widespread with the majority of the participants classified in the poorest socio-economic class. Although socio-economic status was not associated with intimate partner violence, participants in the focus groups reported that poverty was a cause of intimate partner violence because the husband could not provide for the family and this led to endless quarrels and fights[19,40]. Poverty also increases the vulnerability of women to HIV infection because of risky sexual behaviour for monetary gain. In addition, couples may engage in unprotected sex because they cannot afford condoms. Furthermore, poor people may not afford transport to urban areas to do an HIV test[45].

Our household survey did not show an association between antenatal VCT and intimate partner violence. This lack of association could be due to several factors. Due to the very low uptake of VCT among women, our sample lacked the power to detect a significant association between antenatal VCT and intimate partner violence. Secondly, antenatal VCT may be more closely related to intimate partner violence during pregnancy rather than lifetime history of violence. Thirdly, other factors may be more important predictors of antenatal VCT than intimate partner violence[46,47]. Finally, the association between lifetime history of intimate partner violence and antenatal VCT is likely to be complex with considerable variation between women and across settings[29,32,38].

Nevertheless, both the household survey and the focus groups indirectly showed that intimate partner violence was linked to HIV risky behaviours and interventions for HIV prevention. Firstly, we showed that intimate partner violence is strongly associated with sexual coercion. Sexual coercion is one of the mechanisms in which intimate partner violence increases the risk of HIV infection [20,25,26]. Secondly, we reported that multiple partners were the most frequent reason for intimate partner violence[8,25,27]. Thirdly, alcohol was considered to be a major cause of intimate partner violence[14,19,26,37,40,42]. Fourthly, we showed that women fear to test for HIV, disclose HIV results, and request to use condoms because of fear of intimate partner violence[20,21,26,29]. In a study in Kenya, women who had ever used condoms had also experienced more partner violence[28]. Intimate partner violence has also been reported following disclosure of HIV results by women to their partners[30,47-49]. Though we did not inquire into reasons for refusal of sex, it is likely that this could have been related to women's perceptions of their male partners' risk of HIV resulting in violence[14]. Finally, intimate partner violence and HIV may be linked because they share the same underlying factors, notably poverty, alcoholism, and multiple partners[20,31,32].

In conclusion, our study has re-affirmed that intimate partner violence is common and is related to gender inequality, multiple partners, alcohol, and poverty. Accordingly, programmes for the prevention of intimate partner violence need to target these underlying factors. The suggested link between intimate partner violence and HIV risky behaviours or prevention strategies calls for further studies to clearly establish this relationship.

## Competing interests

The author(s) declare that they have no competing interests.

## Authors' contributions

CASK participated in the conception, design, and implementation of the study, statistical analysis, interpretation and drafting of manuscript. JKT participated in study conception and design of the study. TT participated in study conception, design, statistical analysis, interpretation, and drafting of manuscript. KH participated in study conception, design, interpretation and drafting of manuscript. All authors read and approved the final manuscript.

## Pre-publication history

The pre-publication history for this paper can be accessed here:



## Figures and Tables

**Table 1:** Questionnaire on intimate partner violence among 457 women, Mbale district, Uganda in 2003

▪ Has your husband ever beaten you up?
▪ Why did he beat you?
▪ Has your husband beaten you up during the past year?
▪ Have you ever been threatened by a weapon or had a weapon used against you by your husband?
▪ What was the nature of the weapon?
▪ Have you ever been kicked, bitten or hit by your husband?
▪ Have you ever been raped or otherwise sexually abused by your husband?

**Table 2:** Logistic regression of factors associated with lifetime intimate partner violence among 457 women, Mbale district, Uganda in 2003

**Variable**	**Total ** **n (%)**^a^	**Violence** ** n (%)**^b^	**Unadjusted OR (95% CI)**	**Adjusted OR (95% CI)**
Residence				
Urban	124(27)	56(45)	1.0	
Rural	333(73)	193(58)	1.7(1.1–2.5)	4.4(1.2–16.2)
Age of mother				
15 – 24 years	238(52)	119(50)	1.0	
25 – 45 years	219(48)	130(59)	0.7(0.5–1.1)	
Education of mother				
0 – 7 years	324(71)	200(62)	1.0	
8 years or more	133(29)	49(37)	0.4(0.2–0.5)	0.3(0.1–0.7)
Occupation of mother				
Agriculture	400(88)	221(55)	1.0	
Other	57(12)	28(49)	0.8(0.4–1.4)	
Religion of mother				
Christian	180(39)	100(56)	1.0	
Muslim	277(61)	149(54)	0.9(0.6–1.4)	
Socio-economic status				
Poorest	185(60)	102(55)	1.0	
Least poor	122(40)	64(52)	0.9(0.6–1.4)	
Marital status				
Single/widowed	39(09)	19(49)	1.0	
Married/cohabiting	418(91)	230(55)	1.6(0.8–3.3)	
Parity				
Primipara	100(22)	40(40)	1.0	
Multipara	357(78)	209(59)	1.9(1.2–3.1)	
Age of husband				
19 – 24 years	69(21)	36(52)	1.0	
25 years or more	260(79)	143(55)	1.1(0.7–1.8)	
Education of husband				
0 – 7 years	212(60)	135(64)	1.0	
8 years or more	160(40)	68(43)	0.4(0.3–0.6)	
Did mother attend ANC?				
No	15(03)	8(53)	1.0	
Yes	442(97)	241(55)	1.0(0.4–2.9)	
Delivery in a health facility?				
No	241(53)	145(60)	1.0	
Yes	216(47)	104(48)	0.6(0.4–0.9)	
HIV test during last pregnancy?				
No	412(90)	223(54)	1.0	
Yes	45(10)	26(58)	1.2(0.6–2.2)	
Ever discussed HIV with husband?				
No	199(45)	105(53)	1.0	
Yes	243(55)	139(57)	1.2(0.8–1.7)	
Ever used condom with husband?				
No	341(78)	189(55)	1.0	
Yes	96(22)	52(54)	1.0(0.6–1.5)	
Are you satisfied with your marriage?				
No	106(24)	82(77)	1.0	
Yes	327(76)	159(49)	0.3(0.2–0.5)	0.3(0.1–0.7)
Ever discussed your marriage with someone else?				
No	120(27)	63(53)	1.0	
Yes	320(73)	179(56)	1.1(0.8–1.7)	
Does husband have another partner?				
No	177(40)	74(42)	1.0	
Yes	154(35)	107(69)	3.2(2.0–5.0)	
Don't know	111(25)	63(57)	1.8(1.1–3.0)	2.4(1.02–5.7)
^c^Does husband boast of another partner?				
No	91(59)	55(60)	1.0	
Yes	63(41)	52(83)	3.1(1.4–6.7)	
Does husband refuse to provide for your needs?				
No	329(72)	150(46)	1.0	
Yes	128(28)	99(77)	4.1(2.6–6.5)	
Ever slept with another man during your marriage?				
No	436(95)	233(53)	1.0	
Yes	21(05)	16(76)	2.8(1.0–7.7)	

^a ^column percentages

^b ^row percentages

^c ^subset of variable "Does husband have another partner"

**Table 3:** Frequency of lifetime intimate partner violence among 457 women, Mbale district, Uganda in 2003

**Variable**	**Frequency**	**Percent**	**95% CI**
Ever been beaten	170	37.2	31.8 – 42.6
Beaten in past year	62	13.6	10.6 – 16.5
Threatened with weapon	44	9.6	6.6 – 12.7
Kicked, bitten, hit	72	15.8	11.6 – 19.9
Forced sex	169	37.0	31.1 – 42.8
Lifetime intimate partner violence	249	54.3	48.2 – 60.3

**Table 4:** Type of weapon used among 44 women threatened with a weapon in Mbale district, Uganda in 2003

**Variable**	**Frequency**	**Percent**
*Panga*^1^	16	36
Stick	12	27
Knife	10	23
Other^2^	6	14
Total	44	100

^1^large bush knife

^2^Other included chair, pestle, spear

**Table 5:** Association between sexual coercion and physical violence in 457 women, Mbale district, Uganda in 2003

**Variable**	**Total** ** n (%)**^a^	**Sexual coercion** ** n (%)**^b^	**OR (95% CI)**
Ever been beaten?			
No	287(63)	73(25)	1.0
Yes	170(37)	96(56)	3.8 (2.5 – 5.7)
Beaten in the past year?			
No	395(86)	129(33)	1.0
Yes	62(14)	40(65)	3.7 (2.1 – 6.6)
Threatened with a weapon?			
No	413(90)	142(34)	1.0
Yes	44(10)	27(61)	3.0 (1.6 – 5.7)
Kicked, bitten, hit?			
No	385(84)	125(32)	1.0
Yes	72(16)	44(61)	3.3 (1.9 – 5.5)

^a ^column percentages

^b ^row percentages

**Table 6:** Reasons given for intimate partner violence among the 170 women ever beaten in Mbale district, Uganda in 2003

**Reason**	**Frequency**	**Percent**
Husband has another partner	41	24.1
Neglect of housework	23	13.5
Away without husband's permission	22	12.9
Arguments over money or care	15	8.8
Disobedience or disrespect of husband/in-laws	14	8.2
Woman suspected of infidelity	12	7.1
Refused sex	10	5.9
Husband drunk	9	5.3
Other	16	9.4
No response	8	4.7
Total	170	100.0
